# Dual-Pathway Astrocyte Failure in Parkinson’s Disease: Therapeutic Targeting of Nrf2/TFEB Suppression and cGAS-STING/Ferroptosis Activation

**DOI:** 10.1007/s12035-026-06101-6

**Published:** 2026-08-19

**Authors:** Ahmed M. Abdelaziz

**Affiliations:** Department of Pharmacology and Toxicology, Faculty of Pharmacy, Sinai University–Arish Branch, Arish, 45511 Egypt

**Keywords:** Parkinson’s disease, Neurotoxicity, Astrocytes, Alpha-synuclein, CGAS-STING pathway, Ferroptosis, Neuroinflammation, Nrf2, TFEB, Neuroprotection

## Abstract

Parkinson’s disease (PD) is increasingly recognized as a disorder of glial dysfunction, wherein astrocytes transition from homeostatic supporters to active drivers of neurodegeneration. This review synthesizes recent evidence to propose a novel dual-pathway failure model in which internalized alpha-synuclein orchestrates a self-amplifying cycle of astrocytic toxicity. Pathological alpha-synuclein simultaneously suppresses key cytoprotective systems, the Nrf2-mediated antioxidant response and TFEB-regulated autophagy-lysosomal degradation, while hyperactivating neuroinflammatory signaling via NF-κB/MAPK and the recently implicated cGAS-STING axis, triggered by mitochondrial DNA release. This imbalance fosters chronic oxidative stress, proteostatic collapse, and sustained neuroinflammation. Ferroptosis, a form of necrotic cell death characterized by iron dependency and lipid peroxidation, may represent a likely downstream consequence of astrocytic death when protective failure (Nrf2/TFEB suppression) overlaps with toxic activation (cGAS-STING/NF-κB signaling) and disturbances in iron and lipid homeostasis. The concurrent failure of antioxidant defenses and the buildup of labile iron and peroxidizable lipids could establish a conducive environment for ferroptotic membrane rupture, potentially resulting in secondary neuronal damage. This gliocentric model reframes PD pathogenesis as a feed-forward loop of neurotoxicity originating in astrocytic reprogramming. Therapeutically, breaking this cycle via STING inhibition, Nrf2/TFEB activation, and anti-ferroptotic agents represents a promising but still experimental avenue for intervention aimed at restoring astrocyte homeostasis and potentially halting neurodegeneration. However, it is critical to note that the evidence supporting these approaches is derived almost exclusively from preclinical models, with no approved therapies targeting these astrocytic pathways currently available for PD patients.

## Introduction

Parkinson’s disease (PD) is a common neurodegenerative condition associated with aging and represents the leading movement disorder among older adults [1, 2]. Its primary cause is the buildup of mutated alpha-synuclein (α-Syn) within dopaminergic neurons of the substantia nigra pars compacta (SNpc) [1]. This accumulation leads to neurodegeneration in the SNpc through various mechanisms, including oxidative stress, mitochondrial impairment, inflammation, and programmed cell death. Although the fundamental causes of PD remain largely unclear, environmental factors such as pollution, toxins, physical trauma, and insufficient vitamin D may increase disease risk in individuals with genetic susceptibility [3]. Oxidative stress caused by reactive oxygen species (ROS) is a major cause of PD because it causes neuroinflammation and loss of neurons [4]. Consequently, the neuropathology of PD is complex and involves multiple cellular and subcellular disturbances.

Furthermore, PD presents in two main forms: sporadic PD, which is the most common, and familial PD [5]. Clinically, PD is characterized by motor signs including muscle stiffness, tremor at rest, reduced movement (hypokinesia), and a shuffling walk [6]. Additionally, non-motor symptoms such as cognitive decline, sleep disturbances, psychiatric issues, and autonomic dysfunction are common [1]. The progressive degeneration of non-dopaminergic neurons, particularly GABAergic neurons, is fundamentally responsible for the emergence of these non-motor features [1]. PD is most prevalent in individuals over 65 years of age, whereas its prevalence is below 1% in those aged 45–54 [7]. Prevalence reaches 4% in men and 2% in women over 85 [8], and it is expected to double in the coming decades [7, 8]. This age-related link is thought to result from inflammation, oxidative stress, and subsequent neurodegeneration within the SNpc that accompany aging.

In addition to neurons, astrocytes play a significant role in PD neuropathology. Although astrocytes normally offer neuroprotection against oxidative stress through antioxidant and anti-inflammatory mediators [9], microglial signals can convert them into a detrimental, pro-inflammatory A1 phenotype [9]. Astrocytes also express dopamine receptors that influence neuronal activity [9]. However, PD alters normal astrocyte physiology, and genetic mutations can undermine their protective functions, thereby accelerating disease progression [10]. Moreover, astrocyte function in PD is context-dependent and heavily influenced by ATP levels, potentially yielding both protective and harmful effects [11]. Given this complexity and the still-unclear overall contribution of astrocytes to PD, this review focuses on exploring the potential role of α-Syn in modulating astrocyte function in the disease. This review centers on a novel dual-pathway failure model in astrocytes. The first pathway, a protective failure, entails the inhibition of two essential cytoprotective systems, the Nrf2-mediated antioxidant response and the TFEB-regulated autophagy-lysosomal pathway, resulting in oxidative stress, glutathione depletion, and proteostatic collapse [12, 13]. The second one, inflammatory activation, consists of the hyperactivation of neuroinflammatory signaling via NF-κB/MAPK and the cGAS-STING axis following mitochondrial DNA release, which drives chronic inflammation and cellular senescence [14–17]. The convergence of these pathways ultimately triggers ferroptosis, an iron-dependent, lipid peroxidation-driven cell death, as the end-stage mechanism of astrocytic demise [18–20]. This framework reframes PD pathogenesis as a self-amplifying cycle originating in astrocytic reprogramming and guides the therapeutic strategies discussed herein. Among the numerous astrocyte dysfunctions documented in PD, including glutamate excitotoxicity, metabolic dysregulation, circadian disruption, and mitochondrial transfer deficits, why prioritize Nrf2/TFEB suppression and cGAS-STING/ferroptosis activation? This prioritization rests on four evidence-based rationales. First, these pathways signify convergent hubs rather than discrete flaws. Nrf2 and TFEB are principal transcriptional regulators that govern comprehensive networks of antioxidant and autophagic genes, respectively; their inhibition concurrently elucidates various downstream effects (glutathione depletion, proteostasis collapse, and mitophagy failure) that would typically necessitate distinct explanations [12, 13]. Conversely, cGAS-STING activation integrates diverse inflammatory triggers (mtDNA release, ER stress, and lysosomal dysfunction) into a unified interferon response, offering a single upstream target for a broad anti-inflammatory effect [14, 15]. Second, these pathways are druggable with existing or emerging agents. Unlike broad anti-inflammatories such as NSAIDs that failed in PD trials due to lack of specificity, STING inhibitors (H-151), Nrf2 activators (dimethyl fumarate), and TFEB potentiators (trehalose) have entered clinical development for related conditions [21–24]. Third, ferroptosis appears to be a cell death mechanism that can integrate both protective failure and toxic activation as contributing factors; apoptosis, necroptosis, and pyroptosis lack this dual dependency to the same degree, suggesting that ferroptosis may be a particularly relevant endpoint of the proposed vicious cycle [25, 26]. Fourth, genetic data from PD patients directly implicate these pathways: LRRK2 mutations aggravate astrocytic iron dysregulation and vulnerability to ferroptosis, while GBA1 mutations impair lysosomal function upstream of TFEB [27]. Other astrocyte pathways, like lactate shuttle malfunction and AQP4 dysregulation, are significant but serve as downstream effects or concurrent contributors rather than primary regulators capable of reversing numerous diseases when addressed [28, 29]. The recommended paths are selected not due to the irrelevance of others but because they provide the most therapeutic leverage for disrupting the self-amplifying loop at its core.

While earlier reviews have acknowledged astrocyte involvement in PD, a comprehensive framework that explains how their homeostatic functions are simultaneously subverted while chronic neuroinflammation is promoted remains lacking. This review argues that α-Syn does not simply impair astrocytes but actively reprograms them, establishing a pathological positive feedback loop. This review synthesizes evidence showing that the coordinated disruption of the Nrf2 and TFEB pathways, together with hyperactivation of NF-κB and MAPK signaling, makes astrocyte dysfunction a critical amplifier of PD pathogenesis. Finally, this review critically evaluates the therapeutic implications of interrupting this cycle.

## Astrocyte Physiology: An Overview

Astrocytes are star-shaped glial cells that express high levels of vimentin and glial fibrillary acidic protein (GFAP), making up 20–40% of the CNS’s glial population and offering both structural and metabolic assistance to neurons [30, 31]. Two main categories exist: protoplasmic and fibrous astrocytes. Protoplasmic astrocytes, which are most abundant in gray matter, possess specialized structures known as astrocytic end-feet. These end-feet work alongside vascular pericytes to form the blood–brain barrier (BBB) [32]. Fibrous astrocytes, conversely, are primarily located in the brain’s white matter [33].

In addition to these functions, astrocytes play key roles in maintaining fluid homeostasis and vascular integrity, as well as supporting synaptic plasticity and neurotransmitter signaling [34]. They regulate both the excitatory transmitter glutamate and the inhibitory transmitter GABA via uptake and recycling mechanisms [35]. Moreover, astrocytes provide neurons with glutamine, which neurons then convert to glutamate for use in neurotransmission [36]. After signaling ends, astrocytes take up glutamate again and enzymatically convert it back into glutamine via the enzyme glutamine synthase [37]. Interestingly, astrocytic extensions interact with neurons to preserve the structural integrity of the BBB [38]. A notable structural arrangement is the tripartite synapse, which consists of presynaptic and postsynaptic neurons together with adjacent astrocytes [39]. Furthermore, astrocytes have antioxidant mechanisms, such as superoxide dismutase (SOD) and glutathione (GSH), that neutralize damaging reactive oxygen and nitrogen species (ROS and RNS) [40]. This antioxidant function of astrocytes is essential for maintaining the antioxidant capacity of neurons, making up for neurons’ own limited antioxidant enzymes [30]. Astrocytes also produce and release neurotrophic factors, including nerve growth factor (NGF) and brain-derived neurotrophic factor (BDNF), which promote neuronal survival [41]. Additionally, they help prevent excitotoxicity by effectively removing glutamate after signaling ceases [42]. Their range of functions also includes the expression of multiple ion channels such as Na⁺ and K⁺ and aquaporin-4 (AQP4), which control extracellular ion buffering [43]. Lastly, astrocytes influence neuronal metabolism through processes such as the astrocyte-neuron lactate shuttle, energy storage, and waste removal [43].

**Fig. 1:**
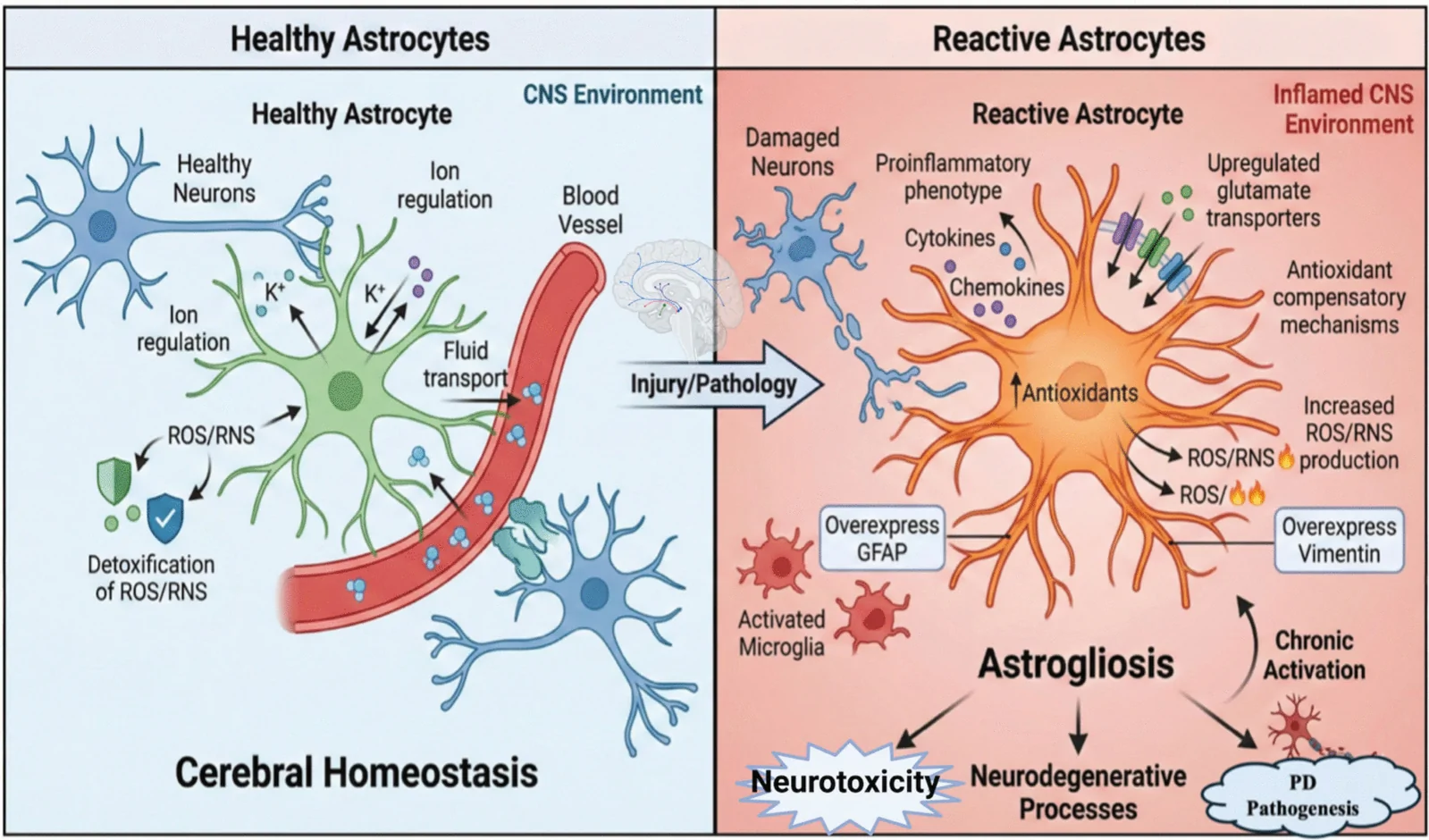
The dual role of astrocytes in brain health and disease: astrocytes play a critical role in maintaining cerebral homeostasis by regulating ions, fluids, and neurotransmitters to support healthy neurons. However, in response to injury or pathology, they transform into a reactive state. This reactive astrogliosis, often triggered by damaged neurons and activated microglia, creates a proinflammatory environment characterized by the release of cytokines, increased oxidative stress, and molecular changes like GFAP overexpression. While initially protective, chronic activation of this state contributes to neurotoxicity and is implicated in the pathogenesis of neurodegenerative diseases like PD. The figure was created using BioRender (Agreement number: SG29Z9QDMK)

Astrocytes are essential for brain health by storing glycogen to modulate neuronal glucose levels [44] and producing nitric oxide (NO) and arachidonic acid (AA) for activity-dependent vasomodulation [45]. Subsequent to brain injury and oxidative stress, astrocytes experience functional and morphological alterations, resulting in reactive astrogliosis to mitigate acute damage (Fig. 1) [46].

### Beyond A1/A2: The Reality of Astrocyte Heterogeneity

The binary classification of astrocytes into neurotoxic A1 and neuroprotective A2 phenotypes has been rendered obsolete by single-cell RNA sequencing (scRNA-seq). In the PD brain, astrocytes exist along a dynamic continuum of states defined by distinct molecular signatures rather than simple polarization. Recent transcriptional atlases have identified specific disease-associated astrocyte (DAA) clusters, such as CHI3L1-high inflammatory astrocytes, which drive neurodegeneration, and Nrf2-high compensatory astrocytes, which attempt to contain damage [47].

Crucially, these states are spatially distinct; astrocytes in the substantia nigra exhibit a profound downregulation of homeostatic genes such as SLC1A2 and KCNJ10 compared to cortical astrocytes, rendering dopaminergic neurons uniquely vulnerable. Therapies must therefore be designed not to flip astrocytes from A1 to A2 but to specifically deplete the CHI3L1 + pathogenic sub-populations while preserving the homeostatic functions of neighboring cells [48].

## Alpha-Synuclein Drives a Vicious Cycle of Astrocytic Dysfunction

It is well established that astrocytes take up pathological α-Syn from stressed neurons, a process that triggers oxidative stress, inflammatory responses, and mitochondrial impairment in these glial cells [49, 50]. Although astrocytes initially serve a protective role by internalizing and breaking down α-Syn much more rapidly than dopaminergic neurons [51], this same function can lead to their dysfunction. This very function becomes a gateway to their own dysregulation. The significance of astrocytes is highlighted by how PD-linked genetic mutations affect them, given their crucial roles in synaptic activity and their capacity for neurogenesis [49, 52]. In addition to direct internalization, astrocytes facilitate the spread of α-Syn by transferring it through tunneling nanotubes and releasing exosomes [53]; these mechanisms can transition from being protective to contributing to disease pathology.

In PD, microglial activation and the resulting neuroinflammation promote the transformation of astrocytes toward pro-inflammatory reactive states, characterized by the secretion of pro-inflammatory cytokines [54]. Single-cell transcriptomic studies have identified specific DAA clusters, such as CHI3L1-high inflammatory astrocytes, that drive neurodegeneration [47]. Thus, suppressing microglial activity can reduce the generation of these pathogenic astrocyte subpopulations in PD animal models [55]. Reactive astrocytes, existing along a dynamic continuum of functional states, engage with endothelial and immune cells amid neuroinflammatory processes [56]. Aggregated α-Syn worsens this situation by entering astrocytes through toll-like receptor 2 (TLR-2), leading to the upregulation of cytokines, including TNF-α and IL-1β [57]. TLR-2 plays an essential role in transmitting the pro-inflammatory effects of α-Syn on reactive astrocytes [58]. Consistent with this disease pathway, astrocytes obtained from individuals with PD exhibit heightened reactivity and greater release of pro-inflammatory cytokines [59]. In astrocytes, aggregated α-Syn triggers both TLR-2 and TLR-4, resulting in the expression of NF-κB and p38 MAPK, as well as enhanced secretion of pro-inflammatory cytokines [58]. Blockade of these TLRs diminishes the α-Syn-evoked cytokine mRNA levels in reactive astrocytes. Increased TLR-4 levels in the SNpc of PD models indicate that extracellular α-synuclein activates astrocytic TLR-4. Moreover, intracellular accumulations of α-Syn within astrocytes inhibit anti-inflammatory factors, namely, Nurr1 [60, 61] and the β-arrestin 2-dependent dopamine D2 receptor (Drd2) [62]. Of note, α-Syn inside neurons similarly degrades Nurr1, exacerbating inflammation and neuronal loss [63, 64]; conversely, agonists targeting Nurr1 alleviate PD symptoms [65]. Activation of Drd2 likewise ameliorates PD manifestations, and Drd2 expression is diminished in PD patients [66]. Astrocytes containing α-Syn aggregates can also function as antigen-presenting cells, engaging with peripheral T cells to foster neuroinflammation in PD [67]. In preclinical studies, α-Syn stimulates astrocytes to express T cell co-stimulatory molecules [67]. Furthermore, genetic alterations such as PARK7 mutations in hereditary PD led to dysregulation of the DJ-1 protein within astrocytes [68, 69]. Under normal conditions, DJ-1 controls the expression of pro-inflammatory genes, including IL-6 and COX-2; when disrupted, this regulation fails, thereby intensifying neuroinflammation and decreasing neurotrophic factor output, which speeds up neurodegeneration [70–73]. In summary, aggregated α-Syn promotes the formation of overactive, pro-inflammatory A1 astrocytes in PD.

### The Cytosolic DNA Sensing Crisis: The cGAS-STING Axis

While classical models attributed astrocytic inflammation primarily to TLR activation, recent breakthroughs identify cytosolic DNA sensing as a more potent driver of the PD inflammatory state. Under homeostatic conditions, mitochondrial DNA (mtDNA) is strictly sequestered within the mitochondrial matrix [14]. However, α-syn oligomers permeabilize the mitochondrial outer membrane in astrocytes, causing the leakage of mtDNA into the cytosol. This ectopic DNA is instantly recognized by cyclic GMP-AMP synthase (cGAS), which synthesizes the second messenger cGAMP [14, 74, 75].

cGAMP binds to the stimulator of interferon genes (STING) on the endoplasmic reticulum, triggering a cascade that is distinct from, yet synergistic with, NF-κB. Activation of STING recruits TANK-binding kinase 1 (TBK1), which phosphorylates interferon regulatory factor 3 (IRF3), driving the transcription of Type I interferons and senescence-associated secretory phenotype (SASP) factors [76–78]. Crucially, recent data from 2024 indicate that this axis is not just inflammatory but metabolic: STING activation in astrocytes suppresses mitophagy, preventing the clearance of the very damaged mitochondria, which leak the DNA. This creates a feed-forward loop where mitochondrial damage begets inflammation, which in turn blocks mitochondrial repair. Targeting this specific axis with STING inhibitors such as H-151 offers a therapeutic precision that broad anti-inflammatories lack [14, 74, 76, 79].

### The Lipid-Invasion Model: ApoE and Lipotoxicity

Astrocytes are the lipid guardians of the CNS, but in PD, this protective capacity transforms into a liability. New evidence suggests a Ping-Pong model of lipotoxicity where stressed neurons, attempting to survive, shuttle toxic peroxidized lipids to astrocytes via Apolipoprotein E (ApoE) particles. Healthy astrocytes sequester these fatty acids into neutral lipid droplets (LDs) for mitochondrial β-oxidation [80, 81].

However, α-syn aggregates inhibit the lipophagy machinery required to break down these LDs, leading to massive intracellular lipid accumulation. These stagnant LDs undergo peroxidation, becoming reservoirs of cellular stress rather than energy depots. This is particularly relevant for carriers of the APOE4 allele, where this transfer mechanism is genetically impaired, leading to accelerated accumulation of toxic lipids in the neuropil [82]. The resulting state is not just metabolic failure but lipotoxicity, where fatty acid overload triggers the NLRP3 inflammasome, directly linking metabolic stress to cytokine release [61, 80, 81, 83, 84].

### The Proteostatic and Metabolic Collapse: From Lysosomal Failure to Energy Crisis

The accumulation of α-Syn in PD stems partly from a failure in astrocytic protein degradation via the ubiquitin–proteasome system (UPS) and autophagy. Although astrocytes are primarily responsible for α-Syn degradation, not transmission [85], this function is impaired in PD, with single-cell studies revealing that specific astrocyte subpopulations exhibit differential capacity for proteostatic maintenance [47]. Supporting this, the natural inhibitor αβ-crystallin, found at higher levels in the SNpc of PD patients, augments α-Syn accumulation in transgenic mice by inhibiting astrocyte autophagy and UPS [86, 87]. Unable to degrade α-Syn effectively, astrocytes instead transfer it to adjacent cells, enabling its spread. This astrocyte dysfunction is consequently a major contributor to PD development and progression.

Additionally, mutant α-Syn accumulation in astrocytes drives energy deficits by causing mitochondrial dysfunction, specifically reducing cellular oxygen consumption [88]. This accumulation also disrupts mitochondrial structure and dynamics through increased ROS production and decreased ATP synthesis [89]. Notably, antibodies targeting astrocytic C-Syn can reverse these mitochondrial defects, restoring both function and morphology [90]. The resulting astrocyte mitochondrial failure reduces ATP supply to neurons, contributing to neuronal damage progression. Dopaminergic neurons in the SNpc are particularly vulnerable, requiring increased oxygen and energy for survival after insults like 6-OHDA exposure [91]. Furthermore, mutant α-Syn accumulation impairs mitophagy, the clearance of damaged mitochondria, in astrocytes, leading to further α-Syn buildup [61, 92]. Astrocytes also play a key role in iron metabolism, expressing all necessary transporters. Importantly, α-Syn disrupts astrocytic iron regulation, partly by dysregulating hepcidin [93]. Excessive iron accumulation in both dopaminergic neurons and astrocytes is strongly linked to advancing neurodegeneration in PD [94].

### A Signaling Imbalance: Subversion of Protection and Amplification of Toxicity

In PD, pathogenic alpha-synuclein orchestrates a profound dysregulation of astrocyte signaling, hijacking critical pathways to drive neurotoxicity. The cytoprotective NRF2 and lysosomal TFEB pathways are suppressed, crippling antioxidant defenses and protein clearance. Simultaneously, alpha-synuclein hyperactivates detrimental signaling cascades. The MAPK pathways, particularly the stress-responsive JNK and p38 branches, and the master inflammatory regulator NF-κB, are potently activated. This signaling imbalance creates a vicious cycle in astrocytes: heightened oxidative stress, impaired lysosomal degradation of pathological proteins, and rampant neuroinflammatory cytokine release. Consequently, astrocytic support functions fail, critically exacerbating neuronal vulnerability and disease progression in the PD brain.

#### NF-κB: The Master Regulator of Neuroinflammation

In PD, the pathologic aggregation of α-Syn not only affects neurons but also potently dysregulates astrocyte function, a process significantly amplified by NF-κB pathway signaling [95]. When astrocytes internalize pathologic α-Syn, particularly oligomeric forms, it triggers the activation of their innate immune receptors, such as TLRs, leading to the canonical activation of the NF-κB transcription factor [96]. This results in the nuclear translocation of NF-κB and the subsequent robust transcription of pro-inflammatory genes, including TNF-α, IL-1β, and iNOS, driving specific astrocyte subpopulations toward a chronically reactive, neurotoxic transcriptional program [47, 97]. This NF-κB–driven astrocytic response creates a self-perpetuating cycle of inflammation that exacerbates neuronal damage, impairs the astrocytes’ normal homeostatic functions like synaptic pruning and metabolic support, and ultimately contributes to the progressive neurodegeneration characteristic of PD [98]. Therefore, the NF-κB pathway acts as a critical molecular amplifier, translating α-syn pathology into sustained astrocyte dysregulation and driving the inflammatory landscape of the PD brain.

#### MAPK Pathway: JNK and p38 as Stress Transducers

The intricate dysregulation of astrocytes, transitioning from crucial CNS supporters to active contributors to pathology, represents a seminal process in PD, with the stress-activated mitogen-activated protein kinase (MAPK) pathways specifically c-Jun N-terminal kinase (JNK) and p38, acting as the primary intracellular signaling nexus that translates the α-Syn insult into a multifaceted astrocytic failure [98, 99]. The cascade is initiated when pathogenic, oligomeric species of α-Syn, either released from degenerating neurons or propagating through the brain’s connectome, engage astrocytic surface receptors such as TLR2/4 or trigger intracellular oxidative stress [100]. This engagement launches a robust phosphorylation cascade, activating upstream kinases such as MKK4/7 for JNK and MKK3/6 for p38, which in turn potently stimulate their respective downstream effectors. The consequences of this sustained JNK and p38 activation are profound and multi-pronged [101–103]. Firstly, it orchestrates a potent and chronic neuroinflammatory response by driving the nuclear translocation and transcriptional activity of pro-inflammatory factors like NF-κB and AP-1 via c-Jun phosphorylation. This results in the rampant synthesis and release of cytokines such as IL-1β, TNF-α,α, and chemokines, creating a self-amplifying inflammatory milieu that directly synaptotoxizes dopaminergic neurons and recruits other immune cells [104]. Secondly, these pathways directly undermine critical astrocytic homeostatic functions; JNK signaling can promote the internalization and degradation of the glutamate transporter EAAT2/GLT-1, leading to excitotoxic glutamate accumulation, while p38 activation disrupts astrocytic metabolism and antioxidant defense mechanisms, leaving neighboring neurons vulnerable to oxidative damage [105, 106]. Furthermore, emerging evidence implicates these kinases in directly exacerbating the α-syn pathology itself, by impairing astrocytic autophagy-lysosomal pathways, thereby allowing toxic aggregates to accumulate, and potentially by modulating the packaging of α-Syn into exosomes for export, thus facilitating the prion-like spread of pathology to healthy cells [107]. Therefore, the JNK and p38 MAPK pathways function as critical signal amplifiers, transforming astrocytes from passive victims of α-Syn pathology into active drivers of a neurodestructive cycle, making them compelling therapeutic targets to neuroinflammatory and progressive neurodegenerative processes in PD.

#### Nrf2 Signaling: The Compromised Guardian

In PD, the pathological misfolding and aggregation of α-Syn initiates a destructive cycle that profoundly dysregulates astrocyte function, shifting these crucial support cells from protectors to contributors of neurodegeneration. Central to counteracting this dysfunction is the transcription factor Nrf2, the master regulator of the cellular antioxidant response [108]. Under conditions of oxidative stress instigated by toxic α-Syn oligomers, Nrf2 dissociates from its cytoplasmic inhibitor Keap1 and translocates to the nucleus [108]. There, it binds to the antioxidant response element (ARE), driving the expression of a wide array of cytoprotective genes. This includes enzymes like HO-1, NADPH quinone dehydrogenase 1 (NQO1), and the subunits of glutamate-cysteine ligase (GCL), the rate-limiting enzyme in glutathione synthesis [109]. By bolstering this antioxidant defense network, Nrf2 activation directly combats the severe oxidative burden within astrocytes, helping to restore their redox balance and protect their mitochondrial integrity.

Beyond managing oxidative stress, the Nrf2 pathway exerts a critical anti-inflammatory influence on α-synuclein–mediated astrocyte dysregulation [110]. Pathological α-Syn potently activates the NF-κB pathway in astrocytes, driving them toward a pro-inflammatory state characterized by the excessive production of cytokines like TNF-α and IL-1β. Nrf2 signaling actively interferes with this process, both directly and indirectly, to suppress NF-κB–mediated neuroinflammation [111]. Consequently, this quelling of the inflammatory response prevents astrocytes from exacerbating the toxic milieu that damages vulnerable dopaminergic neurons. By simultaneously restoring redox homeostasis and damping down chronic neuroinflammation, a robust Nrf2 response helps preserve essential astrocyte functions, including glutamate clearance, metabolic support, and the release of neurotrophic factors [112]. Therefore, the pathological suppression of Nrf2 is a key step in the vicious cycle, and its targeted activation represents a promising therapeutic strategy to reinforce astrocyte resilience.

#### Lysosomal TFEB: The Faltering Clearance Mechanism

**Fig. 2:**
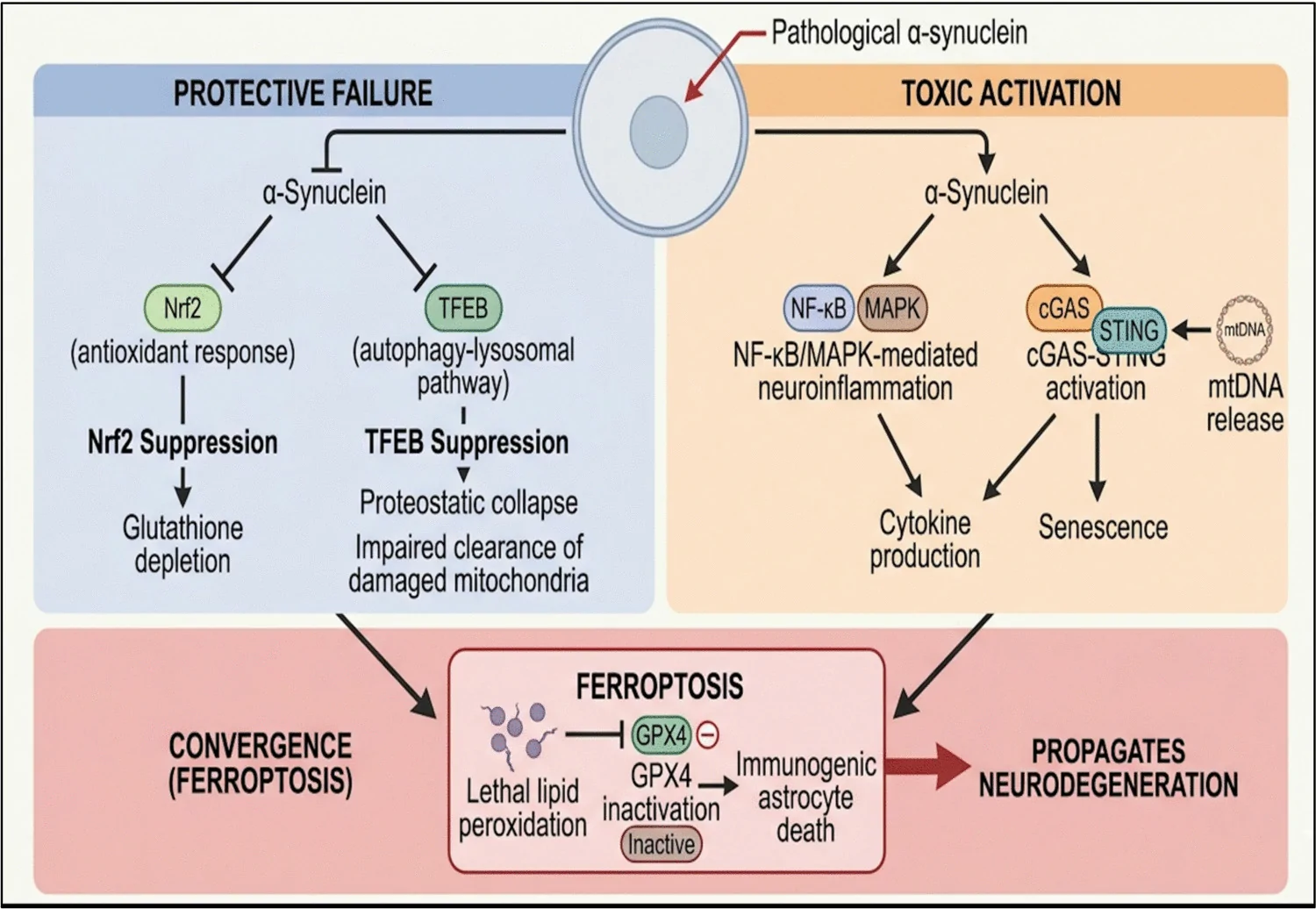
Dual-pathway astrocyte failure in PD. A schematic representation of the dual interrelated processes of astrocytic dysfunction induced by pathogenic α-synuclein. Protective failure (left pathway): α-synuclein obstructs Nrf2 and TFEB, resulting in impaired clearance of injured mitochondria, depletion of glutathione (GSH), and failure in proteostasis. Toxic activation (right pathway): α-synuclein triggers the release of mtDNA, which engages the cGAS-STING axis, while concurrently driving NF-κB/MAPK-dependent neuroinflammation. These pathways operate through parallel and potentially interdependent mechanisms, with emerging evidence suggesting reciprocal activation and positive feedback interactions between cGAS-STING and NF-κB signaling. Together, they promote cytokine production, iron dysregulation, and lipid peroxidation. Ferroptosis (convergent endpoint, bottom): Neither pathway alone is sufficient; ferroptosis occurs only when protective failure depletes GSH (disabling GPX4) AND toxic activation supplies iron and peroxidizable lipids. Their convergence culminates in lethal lipid peroxidation, GPX4 inactivation, and immunogenic astrocyte destruction. The figure was created using BioRender (Agreement number: MO29Z9RMCA)

The insidious accumulation of pathological α-Syn within astrocytes initiates a cascade of dysfunction, corrupting these crucial support cells into a reactive, pro-inflammatory state that exacerbates neurodegeneration [113]. This astrocytic dysregulation is characterized by a crippling impairment of their lysosomal system, the primary machinery for degrading protein aggregates like α-Syn. Enter the transcription factor EB (TFEB), a master regulator of lysosomal biogenesis and autophagy, which represents a powerful endogenous defense mechanism [114, 115]. Under conditions of lysosomal stress, such as that induced by α-Syn overload, TFEB is activated and translocates to the nucleus, where it binds to Coordinated Lysosomal Expression and Regulation (CLEAR) network genes [116, 117]. This orchestrated genetic program dramatically upregulates the synthesis of lysosomal hydrolases, membrane proteins, and components of the autophagic machinery, thereby enhancing the entire autophagic flux from the encapsulation of α-Syn oligomers in autophagosomes to their final degradation within revitalized lysosomes [116, 117]. By clearing this proteotoxic burden, TFEB signaling directly counteracts α-Syn–mediated astrocyte dysregulation: it quells the chronic activation of neuroinflammatory pathways like NF-κB, reducing the secretion of cytotoxic cytokines such as IL-1β and TNF-α, and concurrently helps restore critical homeostatic functions, including synaptic glutamate clearance and the production of neurotrophic factors [118]. Therefore, the strategic pharmacological potentiation of the lysosomal pathway through TFEB activation emerges as a highly promising therapeutic strategy, not merely to clean up cellular debris but to fundamentally reprogram diseased astrocytes, shifting them from drivers of pathology back to guardians of neuronal health in the PD (Fig. 2).

### Critical Perspective: From Linear Causality to a Vicious Cycle

A critical examination of α-Syn–induced astrocyte dysfunction reveals a field grappling with the transition from linear causality to a model of vicious cyclical pathology. The prevailing narrative, which posits that neuronal α-syn passively “spills over” to impair astrocytes, is an oversimplification that ignores the potential for primary astrocytic vulnerability, perhaps via genetic mutations like LRRK2 or GBA1, to initiate or accelerate the disease process [27, 119, 120]. This process is further complicated by methodological constraints, as reliance on in vitro models using high concentrations of pre-formed fibrils fails to replicate the chronic, multicellular environment of the human brain. The astrocytic response is not merely a failure of function but a pathological transformation; the very attempt to phagocytose and degrade α-syn can overwhelm lysosomal systems, triggering inflammasome activation and turning a protective mechanism into a self-destructive one [51, 121]. Moreover, the popular A1/A2 astrocyte dichotomy, while useful, is an inadequate framework for capturing the complex, heterogeneous, and dynamic reactive states in PD, potentially obscuring key pathogenic pathways [122, 123]. This complexity is amplified by a relentless feedback loop, where α-Syn–activated astrocytes release inflammatory signals that drive microglia to produce more toxic cytokines and potentially exacerbate α-Syn pathology, creating an intractable inflammatory circuit [54, 55, 124]. Consequently, therapeutic strategies must evolve beyond simply boosting clearance or suppressing inflammation and instead aim for a nuanced restoration of astrocytic homeostasis, moving past the reductive good-versus-bad cell narrative to target the specific molecular hubs that sustain this destructive cycle.

**Fig. 3:**
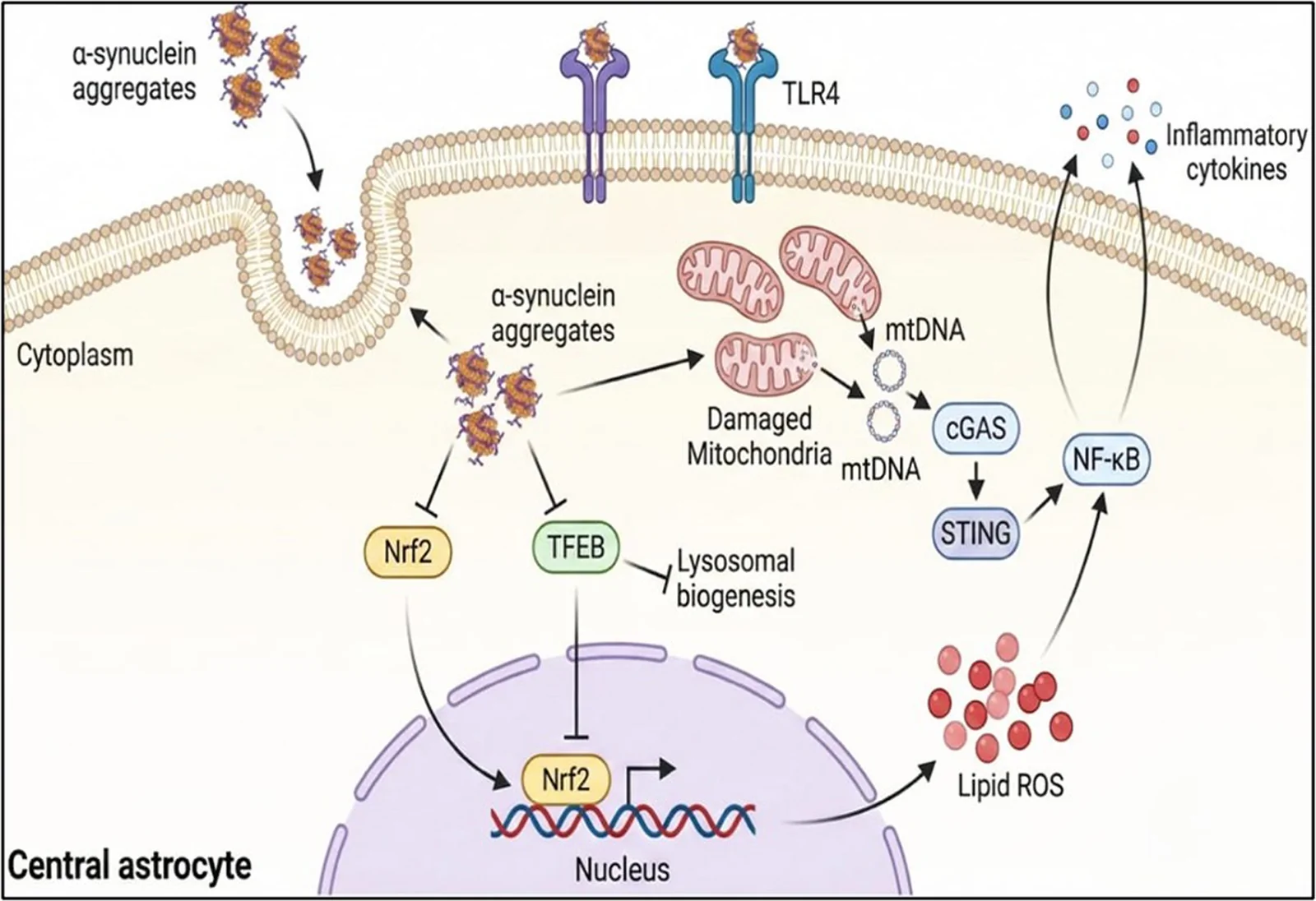
Activation of lysosomal biogenesis for cellular detoxification*.* A schematic of proteostasis and quality control mechanisms in astrocytes. The accumulation of cytoplasmic α-synuclein aggregates and damaged mitochondria triggers the activation of transcription factors Nrf2 and TFEB. Upon translocation to the nucleus, these factors upregulate genes responsible for lysosomal biogenesis, enhancing the cell's capacity for autophagic clearance of toxic debris. The figure was created using BioRender (Agreement number: YL29Z9S8P8)

In summary, the interplay of impaired protein degradation, neuroinflammation, glutamate excitotoxicity, oxidative stress, and mitochondrial failure creates a self-reinforcing vortex of astrocytic dysfunction. This cascade leads to reduced release of neurotrophic factors like BDNF, GDNF, and CNTF, further increasing neuronal injury and exacerbating the core pathologies of PD. This model suggests that astrocyte dysfunction is not merely a secondary response but an integral amplifier that propels PD neuropathology forward (Fig. 3).

### A Potential Convergent Pathway: Astrocyte Ferroptosis

While neuronal ferroptosis is well-characterized, the susceptibility of astrocytes to this iron-dependent cell death has recently emerged as a potentially important contributor to PD progression [18]. Critically, ferroptosis may not be an entirely independent third pathway but rather a potential terminal mechanism that can emerge from the convergence of the two preceding astrocytic failure mechanisms described above [18, 125]. Unlike apoptosis, which preserves membrane integrity, ferroptosis is an immunogenic form of necrotic cell death driven by the lethal accumulation of lipid peroxides, a process normally held in control by the glutathione-dependent enzyme glutathione peroxidase 4 (GPX4) [25, 126]. Protective pathway failure, Nrf2 suppression, depletes GPX4’s essential cofactor glutathione [127, 128], while toxic pathway activation (cGAS-STING/NF-κB) promotes iron dysregulation and lipid peroxidation. Ferroptosis may be most pronounced when both arms converge [25, 26]. In the PD brain, astrocytes face a combination of converging vulnerabilities that may dismantle their defenses, creating conditions permissive for ferroptotic cell death [20, 129].

#### Mechanisms of Susceptibility

First, iron sink failure: Astrocytes normally act as the brain’s iron guardians, sequestering excess extracellular iron via transferrin receptors to protect neurons. However, in PD, α-synuclein aggregates disrupt ferritinophagy (the autophagic degradation of ferritin), releasing labile, reactive iron (Fe^2^⁺) into the cytoplasm. Crucially, recent data reveal that *LRRK2* mutations (G2019S and R1441C) specifically exacerbate this by dysregulating Rab8a/Rab10-mediated iron transport, trapping ferrous iron in lysosomes, and priming astrocytes for oxidative collapse [130, 131].

Second, the antioxidant collapse: The α-synuclein–mediated suppression of Nrf2 critically depletes intracellular glutathione, the essential cofactor for GPX4. Without glutathione, GPX4 cannot neutralize lipid peroxides [129, 132].

Third, the lipid fuel and crosstalk failure: The accumulation of polyunsaturated fatty acids (PUFAs) in astrocytic lipid droplets provides abundant fuel for peroxidation. This vulnerability is compounded by dysfunctional crosstalk with oligodendrocytes. Emerging evidence from 2025 highlights that astrocytic ferroptosis is not an isolated event but is critically modulated by intercellular signaling. A landmark study by Zhang et al. (2025) demonstrated that oligodendrocytes in the PD brain exhibit impaired fibroblast growth factor (FGF) signaling, specifically reducing the secretion of FGF1 and FGF9 [132]. Under healthy conditions, these oligodendrocyte-derived factors bind to astrocytic FGFRs to maintain the expression of the Nrf2/SLC7A11/GPX4 axis. The loss of this trophic support renders astrocytes hypersensitive to lipid peroxidation [83, 132].

#### Consequences: Ferroptotic Propagation

The result is a catastrophic failure of astrocyte integrity. When GPX4 defenses collapse, lipid peroxidation ruptures the astrocyte membrane, releasing not only the cell’s accumulated iron but also pro-inflammatory DAMPs and toxic lipid species into the neuropil. This event triggers a secondary wave of neuronal death, termed ferroptotic propagation, effectively spreading necrosis through the nigrostriatal tract [20, 129]. Thus, ferroptosis may represent a critical point of no return in the astrocytic vicious cycle, a pathway through which the convergence of protective failure and toxic activation could translate into irreversible cell death and secondary neuronal injury. This understanding identifies the SLC7A11-GPX4 axis as a critical, disease-modifying firebreak.

Therapeutic induction of the cystine/glutamate antiporter xCT (SLC7A11), for example, via ceftriaxone, has been shown to specifically restore glutathione synthesis in astrocytes, halt lipid peroxidation, and prevent the secondary loss of dopaminergic neurons in preclinical models [133]. Thus, targeting astrocyte ferroptosis represents a shift from merely supporting neuronal survival to actively preventing the spread of necrotic tissue damage.

## Therapeutic Frontiers: Breaking the Astrocytic Vicious Cycle

Current pharmacological interventions for PD remain largely symptomatic, focusing on dopamine replacement rather than disease modification [134]. Importantly, the therapeutic strategies discussed below remain predominantly in preclinical development. To date, no disease-modifying therapy targeting astrocytic Nrf2/TFEB, cGAS-STING, or ferroptosis has completed clinical trials for PD [15, 26, 128, 135, 136]. The translational gap remains substantial, and the following agents should be interpreted as experimental candidates requiring rigorous clinical validation. However, the identification of the dual-pathway astrocyte failure, characterized by the suppression of protective Nrf2/TFEB axes and the hyperactivation of inflammatory NF-κB/cGAS-STING cascades, unveils a new landscape of therapeutic targets [13, 16, 128, 135, 136]. Strategies that specifically reprogram astrocytes from a reactive, senescence-like state back to a homeostatic phenotype offer the potential to arrest neurodegeneration at its glial source.

### Reactivating the Defensive Shield: Nrf2 and TFEB Agonists

Restoring the lost antioxidant and proteolytic capacity of astrocytes is a primary therapeutic imperative. The Nrf2 pathway, suppressed by α-synuclein, can be pharmacologically reactivated to bolster glutathione synthesis and mitochondrial resilience. Dimethyl fumarate (DMF), an FDA-approved drug for multiple sclerosis, has demonstrated the ability to stabilize Nrf2 in astrocytes, reducing oxidative stress and preventing the A1 phenotypic conversion in PD models [22, 137]. Similarly, ceftriaxone, a beta-lactam antibiotic, functions beyond its antimicrobial activity to upregulate the astrocytic glutamate transporter GLT-1 and the cystine/glutamate antiporter (SLC7A11) via Nrf2 activation [138, 139]. This mechanism directly counteracts glutamate excitotoxicity and provides the cysteine necessary for glutathione synthesis, thereby raising the threshold for ferroptosis. Despite these promising preclinical observations, neither DMF nor ceftriaxone has been evaluated in PD-specific clinical trials. DMF’s FDA approval for MS does not guarantee efficacy in PD, and ceftriaxone’s antibiotic properties limit chronic use. Thus, these agents currently serve as proof-of-concept tools rather than ready-for-translation therapies.

Concurrently, rescuing lysosomal function is critical for clearing α-synuclein aggregates. Small molecule activators of TFEB, such as trehalose and fisetin, promote the nuclear translocation of TFEB, enhancing the transcription of the CLEAR (Coordinated Lysosomal Expression and Regulation) gene network. This reactivation restores autophagic flux in astrocytes, facilitating the degradation of proteotoxic inclusions and damaged mitochondria, mitophagy, before they can trigger cytosolic DNA sensing [140].

### Extinguishing the Inflammatory Core: cGAS-STING and GLP-1R Modulation

While restoring defense is crucial, silencing the aberrant inflammatory signaling that drives neurotoxicity is equally vital. The recent implication of the cGAS-STING axis in astrocytic senescence has highlighted STING inhibitors, such as H-151, as promising candidates. By covalently binding to STING and preventing its palmitoylation, H-151 blocks the downstream interferon response and SASP (Senescence-Associated Secretory Phenotype) release, effectively dampening the neuroinflammatory milieu without compromising broad immune surveillance [14, 141].

Furthermore, interrupting the microglia-to-astrocyte signaling axis can prevent the induction of pathogenic reactive astrocyte states. Glucagon-like peptide-1 receptor (GLP-1R) agonists, particularly NLY01, have emerged as lead candidates in this domain. NLY01 penetrates the BBB and binds to GLP-1Rs on microglia, inhibiting the release of IL-1α, TNF-α, and C1q, a signaling cocktail that drives the conversion of astrocytes toward neurotoxic inflammatory phenotypes [142, 143]. Notably, the efficacy of such interventions may depend on the specific astrocyte subpopulations present, as the sensitivity to microglial signals varies across the heterogeneous astrocyte landscape [47, 48]. Notably, NLY01 represents a rare exception to the predominantly preclinical landscape, having completed a Phase 2 randomized, double-blind, placebo-controlled trial in early untreated PD (NCT04154072) [142]. However, this trial did not meet its primary endpoint of slowing motor progression [142], highlighting the challenges of translating glial-targeted strategies. While other GLP-1R agonists (exenatide, liraglutide) have shown mixed results in PD clinical trials, none are approved for disease modification [144–146]. Thus, despite mechanistic promise, clinical validation remains elusive.

### Preventing the Final Collapse: Anti-ferroptotic Strategies

Finally, preventing the potential necrotic death of astrocytes via ferroptosis could help preserve the structural support of the nigrostriatal tract. Beyond Nrf2 activators, specific iron chelation strategies such as deferiprone can reduce the labile iron pool in astrocytes, preventing the Fenton reactions that drive lipid peroxidation [147]. Emerging preclinical evidence also points to lipophilic radical-trapping antioxidants such as Ferrostatin-1 analogues as potent inhibitors of ferroptotic membrane rupture, offering a direct means to preserve blood–brain barrier integrity and prevent the secondary spread of inflammatory DAMPs [139, 147–149]. Critically, no anti-ferroptotic agent has advanced to clinical trials for PD [149, 150]. Deferiprone, an iron chelator, has been tested in PD (FAIRPARK-II trial) but did not demonstrate significant clinical benefit despite reducing nigral iron deposition [150]. Ferrostatin-1 and its analogues remain strictly investigational, with no human safety data available [149]. The translational barrier for ferroptosis inhibitors is compounded by concerns about chronic iron chelation and off-target effects [147].

### Rationale for Multi-target Combination Strategies: Addressing the Dual-Pathway Failure

Given that the proposed model posits ferroptosis as a potential convergent endpoint that may arise when protective failure (Nrf2/TFEB suppression) and toxic activation (cGAS-STING/NF-κB hyperactivation) overlap with disturbances in iron and lipid homeostasis [18, 25, 26, 127, 128, 132], a compelling question emerges: would simultaneous targeting of both arms yield greater efficacy than single-pathway intervention alone? The theoretical rationale for combination therapy is robust. From a mechanistic standpoint, activating Nrf2 or TFEB alone might replenish glutathione and restore autophagic flux [12, 13, 108, 109, 112, 114–117], yet without addressing the cGAS-STING-driven inflammatory cascade [14–17, 26, 151], ongoing mitochondrial damage and cytokine-mediated suppression of these pathways could potentially undermine their efficacy. Conversely, inhibiting STING alone might block type I interferon signaling and senescence-associated secretory phenotype release [14, 15, 21, 76, 79] but would not directly address the pre-existing proteostatic collapse or glutathione depletion that lowers the ferroptosis threshold [19, 127]. Thus, a dual-pronged strategy, combining a STING inhibitor like H-151 with an Nrf2 activator (e.g., dimethyl fumarate) and/or a TFEB potentiator (e.g., trehalose), could theoretically disrupt the vicious cycle at two independent nodes, potentially achieving synergistic or additive neuroprotection [21, 23, 24, 152]. From a feasibility perspective, several arguments support this approach. First, the proposed agents have distinct, non-overlapping mechanisms of action, reducing the risk of synergistic toxicity. Second, some agents already possess favorable safety profiles in other indications, such as dimethyl fumarate for multiple sclerosis [22, 137] and trehalose as a generally recognized safe compound [23, 24], potentially accelerating repurposing efforts. Third, while the present manuscript does not contain direct PD-specific data for such combinations, preclinical studies in related neurodegenerative conditions have demonstrated that combining an Nrf2 activator with an autophagy enhancer produces greater clearance of protein aggregates than either agent alone, providing proof-of-concept for combinatorial approaches [153]. However, the translational challenges are substantial and must not be understated. The most significant barrier is the complete absence of clinical data for any combination of these agents in PD. No trial has tested, for instance, whether adding a STING inhibitor to an Nrf2 activator improves outcomes compared with monotherapy. The failure of NLY01, a GLP-1R agonist that indirectly suppresses conversion to pathogenic astrocyte phenotypes, to meet its primary endpoint in a Phase 2 trial (NCT04154072) serves as a cautionary tale: even mechanistically rational glial-targeted strategies can fail in humans [142, 145, 154]. Moreover, combination therapy introduces additional complexities: increased pill burden, potential drug-drug interactions, higher costs, and the need for more complex trial designs to detect additive or synergistic effects. Regulatory approval for a combination would require demonstration of superiority over each monotherapy, necessitating large, multi-arm trials that are resource-intensive. Given that no single agent targeting Nrf2, TFEB, cGAS-STING, or ferroptosis has yet demonstrated disease-modifying efficacy in PD patients [150, 151, 153], advocating for combination therapy at this stage remains aspirational rather than evidence-based. The immediate priority must be to determine if any single agent can alter disease progression in rigorous, sufficiently powered clinical trials. Only once such a proof-of-concept is achieved should combination strategies be systematically pursued.

### Additional Therapeutic Strategies

Research has indicated that astrocyte-mediated pathways may represent a promising therapeutic target for neurodegenerative diseases such as PD [155]. Rather than a binary A1/A2 classification, emerging single-cell data reveal that astrocytes exist along a dynamic continuum of reactive states, with specific pathogenic subpopulations (such as CHI3L1-high inflammatory astrocytes) driving neurodegeneration and others maintaining homeostatic functions [47]. Therapeutic strategies should aim to specifically deplete pathogenic subpopulations while preserving neuroprotective astrocyte functions [155]. Several therapeutic strategies aimed at enhancing the neuroprotective functions of astrocytes have been proposed. One key approach involves using viral gene therapy to stimulate astrocytes to release neurotrophic factors like BDNF and GDNF [156, 157]. However, these strategies failed to substantially protect dopaminergic neurons in the SNpc. Additionally, preventing the transformation of astrocytes toward pro-inflammatory reactive states may limit their neurotoxic effects in PD. This transformation is driven in part by microglial activation and the release of specific cytokine cocktails [55]. A preclinical study demonstrated that capsaicin suppresses activated microglia and the release of pro-inflammatory cytokines in rat models of PD, thereby reducing the induction of pathogenic astrocyte phenotypes [158, 159]. Numerous preclinical studies have highlighted that capsaicin also mitigates oxidative stress and neuroinflammation by inhibiting astrogliosis and the release of pro-inflammatory cytokines from activated astrocytes in PD models [160, 161]. Furthermore, lipid-lowering statins have been shown to reduce the emergence of pro-inflammatory astrocyte phenotypes in PD models, thereby reducing PD-related neuropathology [162, 163]. Atorvastatin and simvastatin have also been shown to improve functional outcomes in PD [164]. Astrocyte reprogramming has been found to limit the emergence of neurotoxic astrocyte subpopulations in PD [165]; however, this approach must be carefully calibrated to avoid depleting homeostatic and neuroprotective astrocyte subtypes. Interestingly, transplanting astrocytes into the midbrain can lower the levels of α-synuclein and the inflammation and oxidative stress that go along with it [166]. Consistent with these findings, astrocyte grafting increases dopaminergic neuron density and reduces PD severity in rat models [167]. Thus, the mechanistic roles of astrocytes are diverse, and therapeutic agents can be tailored to target astrocytic pathophysiology. These emerging therapeutic agents and their effects on astrocytic pathophysiology are summarized in Table 1.

**Table 1:** Astrocyte-mediated pathogenic domains and therapeutic opportunities in Parkinson’s disease

Pathogenic domain	Key molecular drivers	Therapeutic strategy	Key preclinical evidence	References
Oxidative stress and iron dyshomeostasis (converge on ferroptosis)	↓ Nrf2/ARE, ↓ GSH, ↓ SOD2; ferritin/FPN1 imbalance, hepcidin ↑	Nrf2 activators (sulforaphane, DMF); iron chelators (deferiprone); hepcidin antagonists	Nrf2 activation protects dopamine neurons in α-Syn models; PD astrocytes retain iron → neuronal vulnerability	[12, 113, 168–175]
Neuroinflammation and microglia-astrocyte crosstalk	NF-κB/STAT3 hyperactivation, ↑ IL-1β/TNF-α; complement (C3/C1q), CX3CL1, P2X7R	NF-κB blockers; GLP-1R agonists (NLY01); complement inhibitors; CX3CL1 modulators	Inhibiting astrocytic NF-κB reduces neuron loss; crosstalk drives dopamine neuron loss in MPTP models	[124, 142, 176–180]
Proteostasis failure (α-Syn and lysosomal dysfunction)	Lysosomal dysfunction (GBA1), defective LAMP1/LCB3; ↑ α-Syn aggregation	Autophagy enhancers (rapamycin, trehalose); aggregation inhibitors (anle138b); GBA1 chaperones (ambroxol)	Astrocytes internalize but fail to degrade α-Syn in advanced PD; mutant astrocytes release more α-Syn in iPSC models	[27, 51, 119–121, 181, 182]
Metabolic and mitochondrial dysfunction	Disrupted lactate shuttle (MCT1/4), glycogen depletion; defective TNTs, ↓ Cx43	Lactate supplementation; ketogenic diets; promote mitochondrial transfer (↑ Cx43)	Astrocytic lactate supports neuronal survival; astrocyte-derived mitochondria rescue neurons in co-culture	[29, 181, 183–187]
Excitotoxicity and BBB disruption	↓ EAAT1/GLT-1 uptake, mGluR activation; ↑ MMP-9, ↓ claudin-5/ZO-1, ↑ VEGF	GLT-1 upregulators (ceftriaxone); mGluR5 antagonists; MMP inhibitors; tight-junction stabilizers	GLT-1 upregulation improves motor function in MPTP mice; astrocytic VEGF correlates with BBB leakage in PD	[105, 188–195]
Loss of trophic support and circadian disruption	↓ GDNF/BDNF, ↓ Wnt/β-catenin; altered BMAL1/CLOCK, ↓ melatonin receptor	GDNF gene delivery; Wnt enhancers; melatonin agonists; circadian modulators	Intrastriatal GDNF shows inconsistent outcomes; astrocytic clocks regulate α-Syn clearance	[196–202]

These findings indicate that targeting astrocytes can attenuate the progression of PD in preclinical models. However, the reader must recognize a fundamental limitation: the therapeutic landscape described above remains overwhelmingly preclinical. With the sole exception of GLP-1R agonists, which have yielded mixed clinical results, none of the discussed agents, including STING inhibitors (H-151), TFEB activators (trehalose and fisetin), Nrf2 activators (DMF and sulforaphane), or ferroptosis inhibitors (Ferrostatin-1 and deferiprone), have demonstrated definitive disease-modifying efficacy in PD patients. Many lack even Phase 1 safety data for this indication. Thus, rigorous preclinical trials and prospective clinical studies are urgently recommended before any translational claims can be substantiated (Table 2).

**Table 2:** Therapeutic targets in the dual-pathway astrocyte failure model of Parkinson’s disease: mechanisms, convergence, and pharmacological modulators

Pathway arm	Key molecular node	PD-associated defect	Consequence of dysregulation	Experimental agent(s)	Developmental stage for PD	Key references
**Protective failure**	Nrf2	Suppressed by α-synuclein; impaired nuclear translocation	Glutathione depletion, oxidative stress, lowered ferroptosis threshold	Dimethyl fumarate, ceftriaxone	Preclinical only	[13, 107, 136, 138, 152]
	TFEB	Impaired nuclear translocation; reduced CLEAR gene transcription	Proteostatic collapse, impaired mitophagy, α-Syn accumulation	Trehalose, fisetin	Preclinical only	[13, 24, 113, 116]
**Toxic activation**	cGAS-STING	Activated by α-Syn-induced mtDNA release	Astrocytic senescence, SASP release, chronic neuroinflammation	H-151	Preclinical only	[14, 15, 21, 75, 151]
	NF-κB	Hyperactivated via TLR2/4 and α-Syn oligomers	TNF-α, IL-1β, iNOS transcription; conversion to pathogenic astrocyte phenotypes	NLY01 (GLP-1R agonist)	Failed Phase 2 trial	[55, 96, 124, 142]
**Convergent endpoint**	Ferroptosis (GPX4/SLC7A11)	Requires GSH depletion (Nrf2 failure) AND iron dysregulation + lipid peroxidation	Immunogenic astrocyte death; ferroptotic propagation to neurons	Deferiprone, Ferrostatin-1	Deferiprone failed FAIRPARK-II	[18, 24, 126, 149]

## Discussion

The pathogenesis of Parkinson’s disease has been dominantly viewed through the lens of the vulnerable dopaminergic neuron, with the progressive loss of nigral cells and the attendant motor deficits defining the disease’s essential character. This review, however, synthesizes a formidable and convergent body of evidence to argue that this neuron-centric narrative is critically incomplete. We posit that astrocyte dysfunction is not a passive bystander effect but an active, propagating engine of disease progression, a concept crystallized in our model of α-synuclein–induced dual-pathway failure. This model reframes PD pathogenesis as a self-amplifying loop within the brain’s homeostatic network, where the very cells tasked with protection are subverted into primary agents of neurodegeneration.

The cornerstone of our thesis is the devastating efficiency with which pathological α-synuclein orchestrates a coordinated assault on astrocytic physiology. It does not merely impair these cells; it reprograms them. On one front, it disables the core defensive systems that maintain cellular and metabolic sanity: the Nrf2-mediated antioxidant response is suppressed, depleting glutathione and crippling the cell’s ability to manage oxidative stress, while the TFEB-led lysosomal and autophagic machinery is hobbled, leading to a catastrophic collapse in proteostasis. This loss of protective capacity creates a permissive environment for damage accumulation. Concurrently, α-synuclein unleashes a destructive gain of function by hyperactivating multiple inflammatory signaling axes. While the roles of NF-κB and p38/JNK MAPK are well-appreciated, the recent emergence of the cGAS-STING pathway as a potent driver of astrocytic senescence and interferon response adds a new layer of complexity and potency to the neuroinflammatory milieu. This pathway, triggered by the cytosolic escape of mitochondrial DNA, creates a feed-forward loop where inflammation begets mitochondrial damage, which in turn fuels more inflammation.

The integration of ferroptosis into this model provides a plausible convergent mechanism for this cycle of dysfunction, though further investigation is needed to establish its primacy. To address a point of potential confusion: ferroptosis may not be a third, entirely independent pathway parallel to the two described earlier. Rather, it may represent a terminal executioner mechanism that is most likely to be triggered when protective failure and toxic activation occur simultaneously, alongside disturbances in iron and lipid homeostasis. The two pathways converge lethally on this iron-dependent, lipid peroxidation-driven cell death. From the protective failure arm, suppression of Nrf2 directly depletes glutathione, disabling the central ferroptosis inhibitor GPX4. The toxic activation arm triggers iron dysregulation induced by α-synuclein through cGAS-STING/NF-κB signaling and leads to the accumulation of peroxidized lipids in stagnant lipid droplets, which together serve as the catalyst and fuel for this process. The necrotic nature of ferroptotic astrocyte death means it is not a silent demise; it is an immunogenic event that releases a flood of damage-associated molecular patterns, iron, and toxic lipids into the extracellular space, directly poisoning neurons and inciting further microglial and astrocytic activation. This ferroptotic propagation represents a final common pathway for the spread of tissue necrosis, explaining the progressive and non-cell-autonomous nature of neurodegeneration in PD.

This refined understanding forces a critical re-evaluation of therapeutic strategy. The compelling preclinical successes of Nrf2 activators, TFEB potentiators, STING inhibitors, and anti-ferroptotic agents underscore that astrocytes are not just involved in PD pathology but are a powerful therapeutic lever. Nevertheless, preclinical progress must be differentiated from clinical translation. The unsuccessful outcomes of previous neuroprotective trials, including GDNF, exenatide, and deferiprone, underscore the stark reality that molecular efficacy observed in rodent models does not ensure therapeutic advantage in humans. At present, no therapies aimed at astrocytic Nrf2, TFEB, cGAS-STING, or ferroptosis have received regulatory approval for PD. The clinical significance of these pathways remains hypothetical, despite being mechanistically persuasive. However, the translation of these findings demands a new level of sophistication. The failure of past neuroprotective trials is a stark reminder that broad-spectrum anti-inflammatories or singular pathway modulators are unlikely to succeed against such a complex, multi-pronged pathology. The future lies in precision glial therapeutics. This entails moving beyond the simplistic A1/A2 dichotomy to target the specific transcriptional regulators of pathogenic astrocyte states, developing astrocyte-specific delivery systems using viral vectors with promoters like GFAP or ALDH1L1, and designing stage-specific combination therapies that simultaneously reinforce faltering homeostatic functions while extinguishing hyperactive inflammatory signals.

Ultimately, this synthesis challenges a fundamental dogma of Parkinson’s disease: the primacy of neuronal initiation. We must seriously entertain the provocative hypothesis that primary astrocytic dysfunction, perhaps driven by mutations in genes like *LRRK2* or *GBA1* that are highly expressed in glia, could be the seminal event that creates a permissive environment for synucleinopathy and neuronal vulnerability. Are astrocytes the first domino to fall? Answering this question requires a concerted shift to human iPSC-derived, region-specific models and advanced organoid systems that can recapitulate the nuanced crosstalk of the human brain. By championing this gliocentric view, we do not seek to diminish the tragedy of neuronal loss but to fully understand its cause. The future of disease modification in Parkinson’s may well depend on our ability to reprogram these pivotal glial cells—to break the vicious cycle and coax them back from drivers of pathology to their intended role as guardians of brain health.

## Limitations and Future Perspectives

While the integrated model of dual-pathway astrocyte failure presented in this review provides a compelling framework for understanding PD pathogenesis, it is crucial to acknowledge the conceptual and methodological frontiers that currently limit our understanding and to map the trajectory of future inquiry. A primary conceptual challenge lies in the regional vulnerability conundrum, the perplexing question of why nigral astrocytes appear uniquely susceptible to losing their homeostatic identity compared to those in other brain regions. This limitation is compounded by our heavy reliance on in vitro models that often utilize cortical astrocyte lineages, potentially overlooking the distinct epigenetic and metabolic landscape of the substantia nigra. Furthermore, the field grapples with a crisis of temporal resolution; the literature presents a largely synchronous view of pathway dysregulation, leaving the critical sequence of events largely unmapped. Specifically, while we propose that ferroptosis may serve as a convergent endpoint when protective failure and toxic activation overlap, it remains unclear which mechanism typically fails first in human disease or whether ferroptosis is consistently the dominant cell death mechanism: whether the collapse of Nrf2 primes the cell for ferroptosis, making it ready to die, or whether cGAS-STING activation acts as the initiating insult that subsequently suppresses protective pathways. This distinction has therapeutic implications: if Nrf2 suppression is the primary event, Nrf2 activators might suffice; if cGAS-STING activation drives both arms, STING inhibitors would be more foundational.

Multiple possible sequences merit examination based on current preclinical evidence. The Nrf2-first model asserts that a decline in astrocytic Nrf2 activity, attributable to age-related factors or genetic predisposition and exacerbated by decades of oxidative stress, establishes a conducive environment for glutathione depletion and iron dysregulation. Subsequently, even minimal accumulation of α-synuclein swiftly induces ferroptosis. This model forecasts that Nrf2 activators will be most efficacious as preventive or early-stage therapies. The cGAS-STING-first paradigm posits that mitochondrial damage and mtDNA release trigger the cascade, leading to NF-κB activation, which directly inhibits Nrf2 production through transcriptional interference, since p65 competes for CREB-binding protein. This strategy would emphasize STING inhibitors as the primary treatment, especially in patients with LRRK2 mutations exhibiting significant mitochondrial dysfunction. Third, the parallel-independent model proposes that protective failure and toxic activation arise from separate α-synuclein species (such as oligomers versus fibrils) engaging distinct receptors (TLR2 versus TLR4), such that the two pathways evolve concurrently rather than sequentially. This scenario would mandate combination therapy from disease onset. Fourth, the microglial-priming model suggests that astrocytic pathway activation is secondary to microglial cytokine release, in which case targeting microglia, rather than astrocytes directly, would be more effective. Distinguishing these models requires time-resolved longitudinal studies in human-relevant systems, including serial PET imaging of astrocytic markers in prodromal PD cohorts and single-cell transcriptomic trajectories from postmortem tissue across Braak stages. This obscures the identification of the optimal, first-in-line therapeutic targets.

A second key limitation of this review and the associated literature is the insufficient focus on how bidirectional astrocyte–microglia crosstalk contributes to astrocyte dysfunction in disease. Current research predominantly adopts a unidirectional perspective: microglia drive the conversion of astrocytes to the pathogenic phenotype through signaling molecules such as IL-1α, TNF-α, and C1q while overlooking the importance of reciprocal signaling. In reality, activated astrocytes release chemoattractants like CCL2 and CXCL10, along with activators such as ATP and HMGB1, which further enhance microglial NLRP3 inflammasome activation and cytokine release, thereby creating a self-reinforcing inflammatory loop. This connection hampers the identification of which cell type’s pathway dysregulation is predominant. For example, α-synuclein–induced activation of astrocytic cGAS-STING may result from both cell-autonomous mtDNA release and the phagocytosis of oxidized mitochondrial fragments obtained from microglia, obscuring the distinction between intrinsic and extrinsic triggers. Likewise, astrocytic Nrf2 suppression may stem from chronic microglial TNF-α exposure rather than direct α-synuclein effects, implying Nrf2 activators could work even if astrocytes are not the main pathological site. The field needs cell-type–specific conditional knockouts like astrocyte-selective STING or Nrf2 deletion in PD models and co-culture systems that mirror the temporal sequence of microglial activation followed by astrocytic transformation. Future reviews should explicitly model these bidirectional interactions instead of treating astrocyte dysfunction as cell-autonomous.

Adding to this complexity are under-explored dimensions, such as the profound modulatory influence of APOE genotype on astrocytic lipid metabolism and neuroinflammatory tone, and the neglected role of circadian biology, where the interplay between core clock genes like BMAL1 and astrocytic Nrf2 activity or neuroinflammation remains a complete mystery. A major challenge that requires clear recognition is the lack of clinical support for the therapeutic targets advocated in this review. The dual-pathway failure model produces testable hypotheses; however, the existing evidence is primarily sourced from rodent models subjected to acute toxin exposure (MPTP, 6-OHDA) or α-synuclein preformed fibrils, which inadequately mimic human sporadic PD; immortalized astrocyte cell lines with modified metabolic profiles; and iPSC-derived astrocytes from limited patient cohorts. None of the discussed interventions, STING inhibition, TFEB activation, or ferroptosis blockade, has demonstrated clinical efficacy in PD. The sole agent to reach Phase 2 testing (NLY01) failed its primary endpoint. This translational gap should moderate enthusiasm and emphasize the necessity of rigorous, sufficiently powered clinical trials prior to substantiating any therapeutic claims.

Addressing these gaps mandates a paradigm shift towards a more precise, human-centric, and dynamic research agenda. The next frontier involves constructing high-resolution, multi-omics atlases of astrocyte states from well-characterized human PD brains across Braak stages, integrating transcriptomic and epigenomic data to reveal the master regulators of pathogenic transformation. In parallel, we must engineer a new generation of humanized models, including region-specific iPSC-derived astrocytes and complex 3D assembloids that recapitulate the multi-cellular ecosystem of the brain, thereby enabling the dissection of cell-autonomous vulnerabilities and non-autonomous pathological crosstalk. Armed with this knowledge, the therapeutic vision must evolve beyond blunt pharmacological tools towards cell-type–specific precision. This entails pioneering astrocyte-targeted gene therapies, using promoters like GFAP to deliver transgenes for a dominant-negative IKKβ to silence NF-κB or a constitutively active TFEB to restore lysosomal function—and developing sophisticated biologics such as bispecific antibodies designed to simultaneously target α-synuclein and engage astrocytic clearance mechanisms.

Finally, our strategic approach must become stage-specific and dynamic. In prodromal stages, the focus should be on preventive reinforcement of astrocytic resilience through Nrf2 activators, while established disease demands a combinatorial blockade of the inflammatory core using STING inhibitors alongside pathogenic astrocyte phenotypes suppressors. For advanced disease, bold replacement strategies, such as the grafting of engineered, neuroprotective astrocyte progenitors, may be necessary to reset the toxic microenvironment. Crucially, we must launch a dedicated effort to contain the propagative damage of the disease by identifying the ferroptotic signals released by dying astrocytes and developing therapeutic “firebreaks” to halt the spread of necrosis. By championing this ambitious agenda, we can transition from merely describing neuroinflammation to actively and precisely reprogramming the gliocentric landscape of PD, finally bridging the formidable gap between preclinical promise and clinical breakthrough.

## Conclusions

In conclusion, the evidence is clear that astrocyte failure is a critical, α-synuclein–mediated mechanism in Parkinson’s disease. The intricate crosstalk between impaired proteostasis, chronic neuroinflammation, and metabolic dysfunction within astrocytes creates a self-perpetuating vicious cycle that is central to disease progression. This refined understanding mandates a paradigm shift from purely neuron-centric therapeutic approaches towards glia-centric strategies. The challenge ahead is no longer merely to confirm the role of astrocytes but to harness this knowledge with sophistication, to develop targeted interventions that can disrupt this vicious cycle, restore astrocytic homeostasis, and, ultimately, alter the progressive course of this devastating disease. Nevertheless, the reader must acknowledge that this vision is still aspirational. The therapy methods examined, although mechanistically persuasive and bolstered by significant preclinical evidence, have yet to exhibit clinical success in PD patients. Recognizing this translational gap is crucial to prevent exaggerating the present condition of the discipline. The primary focus is not on clinical deployment but on thorough validation via meticulously constructed, well-powered trials that explicitly evaluate astrocyte-targeted therapies in human PD. The future of PD therapeutics may well depend on our ability to reprogram these pivotal glial cells from drivers of pathology back to guardians of brain health.

## Data Availability

No datasets were generated or analysed during the current study.

## Abbreviations

AA: Arachidonic acid
AP-1: Activator protein 1
AQP4: Aquaporin-4
ARE: Antioxidant response element
ATP: Adenosine triphosphate
BBB: Blood-brain barrier
BDNF: Brain-derived neurotrophic factor
BMAL1: Brain and muscle ARNT-like 1
Cx43: Connexin 43
Drd2: Dopamine D2 receptor
EAAT1/2: Excitatory amino acid transporter 1/2 (GLAST/GLT-1)
EGF: Epidermal growth factor
GBA1: Glucocerebrosidase gene
GCL: Glutamate-cysteine ligase
GDNF: Glial cell line-derived neurotrophic factor
GFAP: Glial fibrillary acidic protein
GSH: Glutathione
HO-1: Heme oxygenase-1
IGF: Insulin-like growth factor
IL-1β, IL-6, IL-10: Interleukin-1β, -6, -10
iNOS: Inducible nitric oxide synthase
JNK: C-Jun N-terminal kinase
LAMP1: Lysosomal-associated membrane protein 1
LC3B: Microtubule-associated proteins 1A/1B light chain 3B
LRRK2: Leucine-rich repeat kinase 2
MAPK: Mitogen-activated protein kinase
MCT1/4: Monocarboxylate transporter 1/4
MKK3/4/6/7: MAP kinase kinase 3/4/6/7
MMP-9: Matrix metalloproteinase-9
NF-κB: Nuclear factor kappa-light-chain-enhancer of activated B cells
NGF: Nerve growth factor
NO: Nitric oxide
NQO1: NADPH quinone dehydrogenase 1
Nrf2: Nuclear factor erythroid 2–related factor 2
Nurr1: Nuclear receptor related 1 protein
p38 MAPK: P38 mitogen-activated protein kinase
PD: Parkinson’s disease
PINK1: PTEN-induced kinase 1
RNS: Reactive nitrogen species
ROS: Reactive oxygen species
SNpc: Substantia nigra pars compacta
SOD: Superoxide dismutase
STAT3: Signal transducer and activator of transcription 3
TFEB: Transcription factor EB
TGF-α/β: Transforming growth factor alpha/beta
TLR-2/4: Toll-like receptor 2/4
TNF-α: Tumor necrosis factor-alpha
TNTs: Tunneling nanotubes
UPS: Ubiquitin-proteasome system
VEGF: Vascular endothelial growth factor
ZO-1: Zonula occludens-1
α-Syn: Alpha-synuclein
